# AimR Adopts Preexisting Dimer Conformations for Specific Target Recognition in Lysis-Lysogeny Decisions of *Bacillus* Phage phi3T

**DOI:** 10.3390/biom11091321

**Published:** 2021-09-07

**Authors:** Kai Pei, Jie Zhang, Tingting Zou, Zhu Liu

**Affiliations:** National Key Laboratory of Crop Genetic Improvement, Hubei Hongshan Laboratory, Huazhong Agricultural University, Wuhan 430070, China; pk@webmail.hzau.edu.cn (K.P.); zhangjie_guizhou@foxmail.com (J.Z.); zoutingting@mail.hzau.edu.cn (T.Z.)

**Keywords:** phage lysis-lysogeny decisions, arbitrium peptide, protein dynamics, conformational selection mechanism, single-molecule fluorescence resonance energy transfer, small-angle X-ray scattering

## Abstract

A bacteriophage switches between lytic and lysogenic life cycles. The AimR-AimP-AimX communication system is responsible for phage lysis-lysogeny decisions during the infection of *Bacillus subtilis*. AimX is a regulator biasing phage lysis, AimR is a transcription factor activating AimX expression, and AimP is an arbitrium peptide that determines phage lysogeny by deactivating AimR. A strain-specific mechanism for the lysis-lysogeny decisions is proposed in SPbeta and phi3T phages. That is, the arbitrium peptide of the SPbeta phage stabilizes the SPbeta AimR (spAimR) dimer, whereas the phi3T-derived peptide disassembles the phi3T AimR (phAimR) dimer into a monomer. Here, we find that phAimR does not undergo dimer-to-monomer conversion upon arbitrium peptide binding. Gel-filtration, static light scattering (SLS) and analytical ultracentrifugation (AUC) results show that phAimR is dimeric regardless of the presence of arbitrium peptide. Small-angle X-ray scattering (SAXS) reveals that the arbitrium peptide binding makes an extended dimeric conformation. Single-molecule fluorescence resonance energy transfer (smFRET) analysis reveals that the phAimR dimer fluctuates among two distinct conformational states, and each preexisting state is selectively recognized by the arbitrium peptide or the target DNA, respectively. Collectively, our biophysical characterization of the phAimR dynamics underlying specific target recognition provides new mechanistic insights into understanding lysis-lysogeny decisions in *Bacillus* phage phi3T.

## 1. Introduction

During the infection of host cells, temperate phages dynamically switch between lytic and lysogenic life cycles to propagate descendants [1,2,3,4]. In the earlier stage of reproduction, the bacteriophage chose a lytic cycle to replicate the phage DNA, assemble the phage particles, release the virions by lysing the host and start a new infection cycle [5,6]. Later, as the nutrients and the number of the hosts in the environment decreased, the bacteriophage switches to the lysogenic cycle. In the lysogenic cycle, the phage integrates itself into the host genome to propagate along with its host and confers the lysogenized bacteria with immunity against further infection [7,8]. The dynamic transition between the lytic and lysogenic life cycles is vital for phage survival. During the infection, the lysis-lysogeny decisions of the bacteriophage are regulated by an intracellular communication system between co-infecting phages [9,10,11,12].

phi3T phage is a temperate phage, parasitic in *Bacillus subtilis* [13]. Mitomycin C treatment can promote phi3T phage lysis [14], but it was unknown how the phage itself switches between lytic and lysogenic life cycles. In 2017, Zohar Erez and coworkers found an intracellular communication system that controls phi3T phage lysis-lysogeny decisions in the infection of *Bacillus* host cells, by which the descendant phage can communicate with ancestors [10]. This communication system uses an AimR-AimP-AimX axis modulator to determine phage lysis-lysogeny decisions [10,15,16]. This system, named arbitrium system, is encoded by three phage genes, including *aimX*, *aimR* and *aimP*. *aimX* encodes a non-coding RNA that functions as a negative regulator of lysogeny via a yet unknown mechanism. *aimR* encodes a transcription factor that binds to the upstream of *aimX* gene and activates AimX expression, leading to phage lysis. Alternatively, AimR also functions as an intracellular receptor of an arbitrium peptide derived from AimP and transduces this signal into phage lysogeny. The encoded AimP is a pro-peptide with a length of 43 amino acids, which is subsequently secreted out of the infected bacteria. AimP is processed into a mature peptide 6–10 amino acids in length, called the arbitrium peptide. This arbitrium peptide can be internalized by other newly infected bacteria to bind AimR and inhibit its transcriptional activity. Thus, the arbitrium peptide biases the bacteriophages towards lysogeny [10]. Unlike the well-studied communication system of bacteriophage Lambda in the infection of *Escherichia coli* [17,18,19], the molecular mechanism underlying the lysis-lysogeny decisions of *Bacillus* phages is not fully clarified.

The structural bases for the bacteriophage lysis-lysogeny decisions have been extensively studied in SPbeta and phi3T phages [20,21,22,23] (Figure S1). For the SPbeta phage, multiple closed and open conformations of *apo* spAimR dimer have been crystalized, indicating that the spAimR is conformational dynamic (Figure S1a). Crystallography studies showed that the closed and open dimer conformations are responsible for the DNA recognition and SPbeta arbitrium peptide (GMPRGA) binding, respectively [20,21,22,23] (Figure S1a). Mechanistically, our previous single-molecule analysis revealed that in solution, the *apo* spAimR dimer samples inter-conventional closed and open conformations, which allows the DNA or the peptide to selectively bind to a preexisting conformation through a conformational selection mechanism [24]. Alternatively, a dimeric crystal structure of phAimR and a monomeric crystal structure of a phAimR carrying amino acids substitutions in complex with the phi3T arbitrium peptide (SAIRGA) have been determined [21] (Figure S1b). These findings collectively established a proposal that different phages use different mechanisms to regulate the lysis-lysogeny decisions, which is to say, to inhibit the *aimX* activation; the SPbeta arbitrium peptide stabilizes the spAimR dimer, whereas the phi3T arbitrium peptide disrupts the phAimR dimer [21,25,26]. However, how the wild-type phAimR recognizes the phi3T arbitrium peptide and the target DNA in solution is unclear.

Here, we quantitatively characterized the mechanism of phAimR underlying the recognition of the arbitrium peptide and the target DNA in solution. Our gel-filtration, SLS and AUC results show that phAimR is dimeric in solution regardless of the presence of arbitrium peptide. By performing SAXS analysis, we find the binding of the arbitrium peptide makes an extended dimeric conformation. Mechanistically, smFRET analysis reveals that the phAimR dimer fluctuates between two distinct conformational states, and each preexisting sate is selectively recognized by the arbitrium peptide or the target DNA, respectively. The revealed conformational dynamics of phAimR show that it does not undergo dimer-to-monomer conversion to recognize the peptide. It is the intrinsic dynamics of phAimR dimer that allows it to use a conformational selection mechanism to recognize a specific target.

## 2. Materials and Methods

### 2.1. Protein Preparation

The codon-optimized complementary DNA of AimR from Phi3T phage (GenBank: KY030782.1) was subcloned into the pET15b vector (Invitrogen, Shanghai, China) with a N-terminal 6× His tag. The phAimR clone was transformed into *Escherichia coli* strain BL21 (DE3) and induced with 0.2 mM isopropyl β-D-thiogalactopyranoside at an absorbency (600 nm) of 1.1. After induction at 16 °C for 16 h, the cells were collected and resuspended in a buffer containing 25 mM Tris–HCl (pH 8.0) and 150 mM NaCl.

Following further disruption by a homogenizer (JNBIO, Juneng & Bio Technolog, Guangzhou, China), cell debris was removed via centrifugation at 23,000× *g* for 1 h. The supernatant was collected and loaded onto Ni^2+^-nitrilotriacetate affinity resin (Ni-NTA, Qiagen, Shanghai, China), The phAimR was eluted using a buffer containing 25 mM Tris–HCl (pH 8.0), 150 mM NaCl and 250 mM imidazole. The target protein was further purified using anion-exchange chromatography (Source 15Q 10/100, GE Healthcare, Shanghai, China). After the removal of the 6× His tag using drICE protease, phAimR was further purified using size-exclusion chromatography in a buffer containing 25 mM Tris–HCl (pH 8.0), 300 mM NaCl and 5 mM dithiothreitol (DTT). The phAimR carrying amino acids substitutions were generated using a two-step PCR strategy and were prepared in the same way as the wild-type protein.

### 2.2. Gel-Filtration Assay

8.2 μM phAimR, 8.2 μM phAimR in complex with 82 μM SAIRGA peptide, 8.2 μM phAimR^Y341A/E371A^ and 8.2 μM phAimR^Y341A/E371A^ in complex with 82 μM SAIRGA peptide were prepared separately. Each sample was then injected into a Superdex 200 Increase 10/300 GL column equilibrated with a buffer containing 25 mM Tris-HCl (pH 8.0) and 300 mM NaCl. Peak fractions were collected and transferred for SDS-PAGE visualization.

### 2.3. Static Light Scattering Experiment

phAimR or phAimR^Y341A/E371A^ was prepared at 1.6 mg/mL, and for the preparation of the peptide-bound form, a 10-fold molar excess of SAIRGA was incubated. Each sample was independently loaded onto a Superdex 200 increase 10/300 column connected to a HELEOS multi-angle light scattering instrument (WYATT Technology, Santa Barbara, USA). The protein was eluted in a buffer of 25 mM Tris-HCl (pH 8.0) and 300 mM NaCl at a flow rate of 0.4 mL/min. Each fraction was automatically analyzed using multi-angle light scattering.

### 2.4. Analytical Ultracentrifugation Experiment

An analytical ultracentrifugation experiment was performed in a Beckman Coulter XL-I analytical ultracentrifuge using two-channel centerpieces. A total of 0.64 mg/mL phAimR and SAIRGA peptide-bound phAimR (a 10-fold molar excess of peptide was used) were prepared in a buffer of 25 mM Tris-HCl (pH 8.0) and 300 mM NaCl, respectively. Data were collected via absorbance detection at 18 °C for protein and a rotor speed of 55,000 r.p.m. The SV-AUC data were globally analyzed using the SEDFIT program and were fitted to a continuous c(s) distribution model to determine the molecular weight. The same experiments were performed for phAimR^Y341A/E371A^.

### 2.5. Small-Angle X-ray Scattering Measurement

phAimR and phAimRY^341A/E371A^ were prepared at 100 μM in a buffer containing 25 mM Tris-HCl (pH 8.0) and 300 mM NaCl with or without 200 μM SAIRGA peptide, respectively. SAXS data were collected at the SSRF using the BL19U2 beamline at room temperature. For each measurement, 20 consecutive frames with 1 s exposure time were recorded. SAXS data were averaged after checking that there was no difference between the first and last frames and were processed using the RAW software (bioxtasraw.sourceforge.net, 1.4.0, San Diego, CA, USA). The background scattering was recorded for the matching buffer and subtracted from protein scattering data. The paired distance distribution probability function was obtained from scattering intensity function I(q) using GNOM [27].

### 2.6. Fluorescent Dye Conjugation

The cysteine substitutions of phAimR (phAimR^C6S/C74S/C99S/C136S/C223S/C267S/N103C^ or phAimR^C6S/C74S/ C99S/C136S/C223S/C267S/D174C^) were generated with a standard PCR-based strategy. Proteins were prepared in the same way as the wild-type phAimR. Alexa Fluor 488 C5 maleimide (Alexa488, A10254, Thermo Fisher, Shanghai, China) and Cy5 maleimide (Cy5, PA15131, GE Healthcare, Shanghai, China) were used as fluorescence donor and acceptor, respectively. A desalting column (HiPrep 26/10, GE Healthcare, Shanghai, China) was used to exchange the protein buffer into the conjugation buffer containing 20 mM phosphate (pH 7.4) and 100 mM NaCl. The protein fraction was collected in premixed Alexa488 and Cy5 dyes. The conjugation of phAimR was performed with Alexa488 and Cy5 at a molar ratio of 1:4:4. Conjugation reaction was performed for 3 h at room temperature in the dark, and the mixture was then purified using a Source-Q column (GE Healthcare, Shanghai, China). The fractions of double-labeled protein that had absorption at 280 nm, 493 nm and 640 nm were collected for smFRET data collection.

### 2.7. Single-Molecule Fluorescence Resonance Energy Transfer Measurement

The laser confocal fluorescence microscope MicroTime 200 (PicoQuant, Berlin, Germany) was used for single-molecule imaging, and a pulsed interleaved excitation (PIE) scheme [28] was used with two SPCM-AQRH detectors (Excelitas, Waltham, MA, USA) for recording fluorescence time traces at two different wavelengths. Two picosecond-pulsed diode laser heads (LDH-P-C-485 and LDH-P-C-640; PicoQuant, Berlin, Germany) were driven by a PDL 828 Sepia II driver (PicoQuant) at a repetition rate of 40 MHz, which allowed interleaved excitations for Alexa488 and Cy5. Each laser was coupled to the inverted microscope IX 73 (Olympus, Beijing, China) through a single-mode fiber, and was reflected by a dichroic mirror through a water-immersion objective (UPLSAPO 60×, N.A. 1.20). The protein sample was loaded onto a hybridization chamber (Thermo Fisher) glued to a glass coverslip (Thermo Fisher, Shanghai, China). The laser confocal point was set to about 50 μm above the coverslip. The excitation power at the back of the objective was about 100 μW for the 485 nm laser and about 35 μW for the 640 nm laser, as estimated with a power meter (PM20-FC; Thorlabs, Shanghai, China). Focused to a 100 μm pinhole, the fluorescence emission from the excited protein molecule was collected with the same objective. The donor and acceptor emissions were separated with a dichroic mirror (T635lpxr; Chroma, Xiamen, China). The donor emission was filtered with a 520/35 BP band pass, and the acceptor emission was filtered with a 690/70 BP band pass, before being focused onto the two SPCM-AQRH detectors.

The fluorescence outputs were recorded with a TimeHarp 260 PCI board (PicoQuant, Berlin, Germany) built into a PC workstation, and the data were stored in the time-tagged time-resolved module (PicoQuant, Berlin, Germany). The photon counts including f_Dex/Dem_, f_Dex/Aem_ and f_Aex/Aem_ were obtained by binning the photons in 1 ms bins using SymPhoTime64 software (PicoQuant, Berlin, Germany). Here, f_Dex/Dem_ represents the photon count for the donor excitation and donor emission channel; f_Dex/Aem_ represents the photon count for the donor excitation and acceptor emission channel, and f_Aex/Aem_ represents the photon count for the acceptor excitation and acceptor emission channel. With all three fluorescence time traces recorded, the signals from the donor-only or acceptor-only protein molecules were filtered out, so as to ensure that an f_Dex/Aem_ photon only arises from a doubly labeled protein. A burst search was performed using a start/stop criterion as described [29,30].

The parameters for instrumentation and fluorophores were calibrated following the established protocol [29]. Double-stranded DNA oligonucleotides with different donor–acceptor distances, that is, the fluorophore conjugation sites separated by different numbers of bases (Figure S2), were used to determine the detection correction factor γ and the cross-talk terms (Figure S3). The cross-talk terms included the donor emission detected by the acceptor channel (donor leakage; abbreviated Lk) and the acceptor emission excited by the donor excitation wavelength (acceptor direct excitation; abbreviated Di). Alexa488 was conjugated to the C6-amino group of a dT nucleotide at the 5′ end of one oligonucleotide strand, whereas Cy5 was conjugated to the C6-amino group of an internal dT nucleotide of another oligonucleotide strand. The fluorophore-conjugated oligonucleotides were purchased from Sangong Biotech and further purified using a Source Q column. The purified single stranded DNA oligonucleotides were mixed at room temperature in a buffer containing 40 mM Tris-HCl (pH8.0) and 500 mM NaCl, heated to 95 °C for 2 min and gradually cooled down to room temperature in the dark for annealing. To ensure complete hybridization for the acceptor-labeled DNA strand, the donor-labeled strand had 50% molar excess (the donor-only double-stranded DNA could be filtered out using the PIE scheme). To prepare the donor-only and acceptor-only double-stranded DNA, a 10-fold excess of unlabeled DNA strand was used for annealing.

The smFRET measurements of phAimR were performed at 25 °C in 20 mM Tris-HCl (pH 7.5) buffer, containing 100 mM NaCl, 0.005% (vol/vol) Tween 20 (Thermo Fisher, Shanghai, China), 1 mM L-ascorbic acid and 1 mM methylviologen (Sigma-Aldrich, Shanghai, China). The concentration of the doubly labeled sample was about 100 pM. The smFRET data were collected for about 1 h. The threshold for photon count traces f_Dex/Dem_, f_Dex/Aem_ and f_Aex/Aem_ was 3 to 7 counts per bin depending on the background dark counts. To be classified as a burst, the total photon counts (f_Dex/Dem_ + f_Dex/Aem_) in the burst had to be at least 25 above the background threshold. The exact FRET efficiencies were calculated based on our calibrated parameters for the instrument and fluorophores, and the FRET efficiency distribution was analyzed with a multi-Gaussian mixture using our previously handwritten script [31].

### 2.8. Electrophoretic Mobility Shift Assay

DNA probes were annealed using boiling water with FAM-labeled primers to generate the 41 bp DNA fragment (70262-70302, 5′ GAAATGTCCAGAAATTCAAAAATCAAAAAATAAGAACATGG 3′) from phi3T bacteriophage genome. The labeled probes (5 nM) were incubated with 0, 0.037, 0.11, 0.33 and 1 M phAimR proteins or peptide-bound phAimR complex (peptide is 10-fold molar excess of phAimR) in a buffer containing 25 mM Tris-HCl (pH 8.0), 5 mM MgCl_2_, 5 mM DTT, 0.1 mg·mL^−1^ BSA, 10% glycerol and 150 mM NaCl at room temperature for 10 min. The reactions were resolved on 6% native acrylamide gels (37.5:1 acrylamide:bis-acrylamide) in 0.5× TBE buffer at 100 V for 75 min. Images of the gels were obtained using FLA5100 (Typhoon, Shanghai, China).

## 3. Results and Discussion

### 3.1. phAimR Is Dimeric in Solution Regardless of the Presence of Arbitrium Peptide

**Figure 1:**
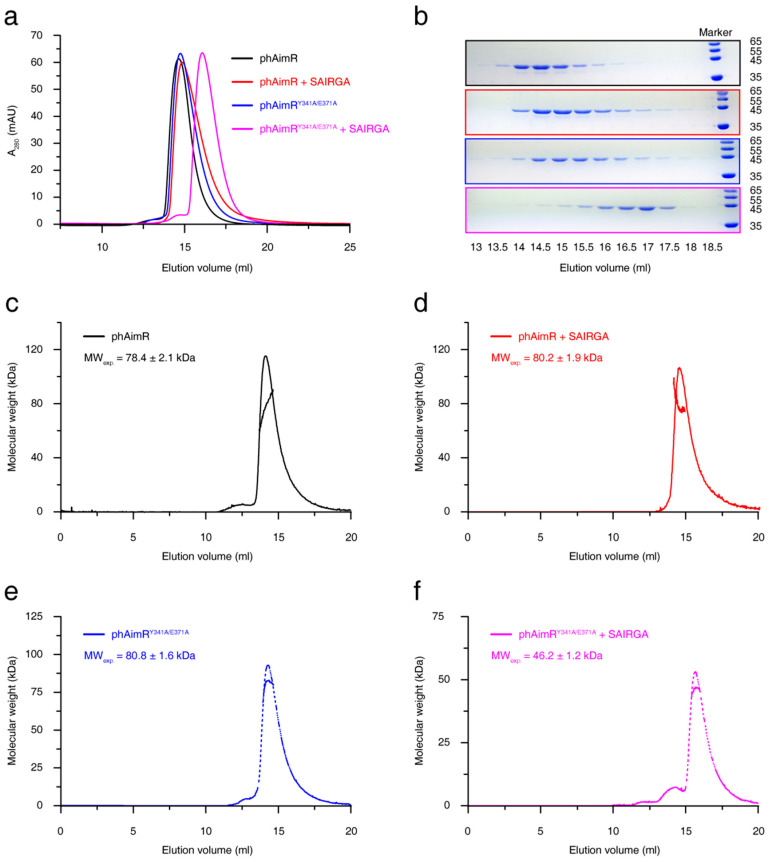
Solution characterization for the changes in the oligomerization states of phAimR upon SAIRGA peptide binding. (**a**) Gel-filtration analysis assesses the effect of SAIRGA peptide binding on phAimR and phAimR^Y341A/E371A^. (**b**) SDS-PAGE gels visualize the elution volume of the peak fractions in (**a**). SLS evaluates the experimental molecular weight of (**c**) phAimR, (**d**) phAimR in complex of SAIRGA peptide, (**e**) phAimR^Y341A/E371A^ and (**f**) phAimR^Y341A/E371A^ in complex of SAIRGA peptide, respectively.

phAimR carrying two amino acids substitutions (phAimR^Y341A/E371A^) has been engineered to crystallize the complex of phAimR and the phi3T-derived SAIRGA peptide, and a monomeric conformation of this complex was determined [21]. Consistent with this, by performing gel-filtration assay using Superdex 200 Increase 10/300 GL column, we found that, in the presence of SAIRGA peptide, the peak of phAimR^Y341A/E371A^ migrated later than the phAimR^Y341A/E371A^ alone peak (Figure 1a,b). In contrast, the SAIRGA peptide binding of the wild-type phAimR produced no significant migration differences (Figure 1a,b). These results indicate that the phAim^Y341A/E371A^ undergoes a dimer-to-monomer conversion upon peptide binding, whereas the wild-type phAimR adopts a dimer conformation regardless of the presence of peptides.

To more quantitatively characterize the changes in the oligomerization states of phAimR upon SAIRGA peptide binding, we further performed SLS analysis. The measured experimental molecular weight of phAimR is 78.4 ± 2.1 kDa, which is about twice its theoretical mass (44.3 kDa) (Figure 1c). For phAimR in the complex of SAIRGA peptide, the experimental molecular weight is 80.2 ± 1.9 kDa, which is also about twice their theoretical mass (44.6 kDa) (Figure 1d). Thus, the phAimR remains a dimer after peptide binding. By contrast, although the phAimR^Y341A/E371A^ is dimeric in solution, the binding of the SAIRGA peptide induced a monomeric conformation (the experimental molecular weight is reduced from 80.8 ± 1.6 kDa to 46.2 ± 1.2 kDa) (Figure 1e,f). We next further validated these results using an AUC experiment. They also showed that the peptide binding of wild-type phAimR made no significant differences in molecular weight, whereas the peptide binding of phAimR^Y341/E371A^ reduced the molecular weight by about 50% (Figure S4). Taken together, phAimR adopts a dimer conformation in solution to recognize the phi3T-derived peptide that is similar to the binding model of spAimR and the SPbeta-derived peptide [20,21,22,23]. The Y341A/E371A double substitutions may produce perturbations in the quaternary structure of phAimR in the recognition of the arbitrium peptide.

### 3.2. phAimR Recognizes Arbitrium Peptide in an Extended Dimer Conformation

**Figure 2:**
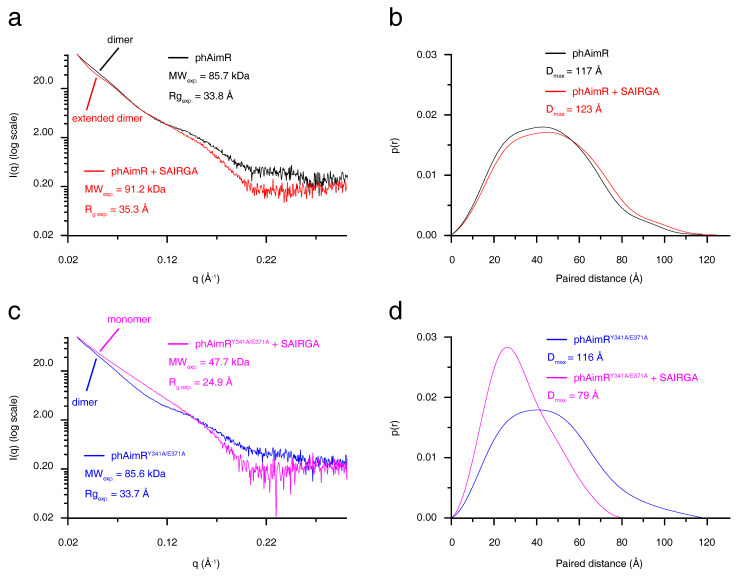
SAXS characterization for the conformational changes of phAimR upon SAIRGA peptide binding. The SAXS profile is shown as (**a**,**c**) the scattering intensity over scattering angle and (**b**,**d**) the paired distance probability. SAXS profile for phAimR, phAimR in the presence of SAIRGA peptide, phAimR^Y341A/E371A^ and phAimR^Y341A/E371A^ in the presence of SAIRGA peptide is colored in black, red, magenta and blue lines, respectively.

To characterize the conformational change of phAimR upon SAIRGA peptide binding, we next performed SAXS analysis. The SAXS-measured experimental molecular weight of phAimR and phAimR in complex with SAIRGA peptide is 85.7 kDa and 91.2 kDa, respectively (Figure 2a). This indicates that phAimR adopts a dimer conformation in solution regardless of the presence of the SAIRGA peptide. It is in line with our gel-filtration, SLS and AUC results (Figure 1 and Figure S4). Structurally, by the binding of a SAIRGA peptide, the experimental radius of gyration (Rg) and the maximum paired distance (D_max_) of the phAimR dimer increased from 33.8 Å to 35.3 Å (Figure 2a) and from 117 Å to 123 Å (Figure 2b), respectively. This increased molecular shape indicates that phAimR recognizes the SAIRGA peptide using an extended dimer conformation.

We also characterized the conformational change of the phAimR^Y341A/E371A^ upon SAIRGA peptide binding (Figure 2c,d). By the binding of the SAIRGA peptide, the measured experimental molecular weight, Rg and D_max_ of phAimR^Y341A/E371A^, decreased from 85.6 kDa to 47.7 kDa, from 33.7 Å to 24.9 Å and from 116 Å to79 Å, respectively. These analyses reveal that the binding of the SAIRGA peptide disassembles the dimeric phAimR^Y341A/E371A^ into a monomeric conformation, in line with previous reports [21].

### 3.3. phAimR Dimer Adopts Two Preexisting Conformational States for the Recognition of Arbitrium Peptide and Target DNA

**Figure 3:**
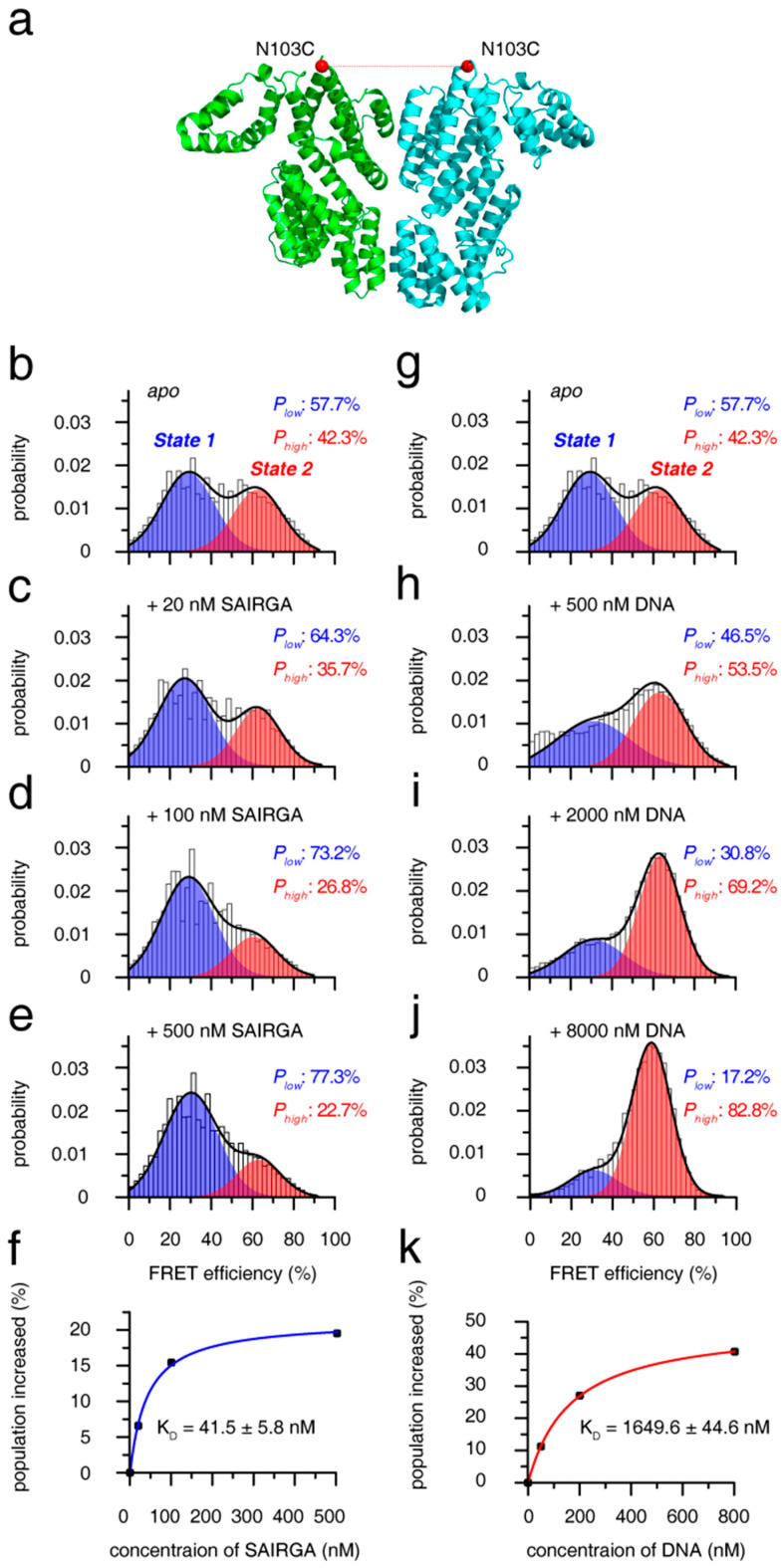
Mechanism of phAimR dimer for the recognition of SAIRGA peptide and target DNA. (**a**) Cartoon representation of phAimR dimer for the fluorophore conjugation site. Structure with 5ZVV PDB code is used for the representation, and the Cα atoms of the N103 residues are shown as red spheres. (**b**–**e**) The change of smFRET profile of phAimR dimer upon SAIRGA peptide titration. The low- and high-FRET species are colored in blue and red, respectively. The SAIRGA peptide selectively enriches the low-FRET species. (**f**) Binding affinity of phAimR towards SAIRGA peptide. (**g**–**j**) The change of smFRET profile of phAimR dimer upon target DNA titration. The DNA selectively enriches the high-FRET species. (**k**) Binding affinity of phAimR towards DNA. Note that panels b and g are identical for easy comparison.

To uncover the mechanism for the recognition of arbitrium peptide and target DNA by phAimR dimer, we further performed smFRET analysis. By substituting all solvent-exposed six intrinsic cysteine to serine (C6S/C74S/C99S/C136S/C223S/C267S) and generating an additional cysteine at the solvent-exposed N103 residue, we specifically labeled phAimR with a fluorescent probe of Alexa488 or Cy5 though thiol-maleimide chemical conjugation (Figure 3a). As there is only one fluorescent probe labeled with phAimR (at N103C position), the observed FRET comes from the phAimR dimer. The smFRET profile of phAimR dimer can be clearly fitted into two FRET species, with low- and high-FRET efficiencies centered at about 30% and 62% and with respective population of ~58% and ~42%, respectively (Figure 3b). FRET efficiency is distance- and structural-dependent; thus, this smFRET result indicates that the phAimR dimer exists in two distinct conformational states in solution. We name the low- and high-FRET species conformational state 1 and 2, respectively, for discussion.

To assess the two conformational states of the phAimR dimer for target recognition, we titrated the phi3T-deriveed SAIRGA peptide into the solution and monitored the changes in the smFRET profile. We found that the SAIRGA peptide selectively enriched state 1 and that the population of state 2 was reduced (Figure 3b–e). Little fluctuation in the low-FRET efficiency during the peptide titration indicates that state 1 counts as a preexisting conformation for peptide recognition (Figure 3b–e). Tracking the increased populations of state 1 by SAIRGA peptide titration, the binding isotherm can be fitted to a K_D_ value of 41.5 ± 5.8 nM (Figure 3f). As a negative control, the SPbeta-derived GMPRGA peptide made no perturbation on the smFRET profile of phAimR (Figure S5a), indicating that there is no interaction between the phAimR and the SPbeta-derived arbitrium peptide, which is consistent with previous reports [10].

The target DNA of phAimR in phi3T phage has been mapped [22]. Here, we also confirmed this interaction between phAimR and the target DNA using electrophoretic mobility shift assays (EMSA) and reveal that only the phi3T-derived SAIRGA peptide, but not the SPbeta-derived GMPRGA peptide, can abolish the DNA binding ability of phAimR (Figure S6). Next, we assessed the mechanism of the phAimR dimer for the target DNA recognition by performing an smFRET titration experiment. The smFRET results showed that preexisting conformational state 2 is selectively enriched at the expense of state 1, and the binding isotherm can be fitted to a K_D_ value of 1649.6 ± 44.6 nM (Figure 3g–k). The ability of phAimR binding to the target DNA revealed at single-molecular level is about 40-fold weaker than that to the phAimR-derived peptide (Figure 3f,k). This is similar to spAimR, that is the binding of spAimR to its target DNA, (K_D_ = 101.5 nM) is much weaker than that to the SPbeta-derived peptide (K_D_ = 4.6 nM) [20]. We also collected the fluorescence correlation spectroscopy (FCS) data of the phAimR dimer at single-molecular level and analyzed the changes of the dual color cross-correlation function *G*_AD_ (τ) upon the binding of the SAIRGA peptide and target DNA. The profile of cross-correlation function *G*_AD_ (τ) is molecular-shape-dependent [32,33,34], thus the observed different *G*_AD_ (τ) profiles of phAimR in the presence of SAIRGA peptide and DNA indicating that the phAimR dimer adopts different conformation to recognize these two targets (Figure S7). This is consistent with our smFRET findings that the different conformational states 1 and 2 are responsible for peptide and DNA recognition, respectively (Figure 3). Taken together, the quaternary dynamics of the phAimR dimer enable it to recognize phi3T-derived peptide or target DNA using a conformational selection mechanism, in which one preexisting state is enriched and stabilized, and the other state is interconverted towards this preexisting state via an equilibrium shift.

To validate our smFRET findings, we further prepared dyes labeled phAimR with another labeling site (solvent-exposed D174C) and performed smFRET analysis (Figure S8a). Results shows that there are also two FRET species, with low- and high-FRET efficiencies centered at about 35% and 63%, respectively (Figure S8b). The population of low- and high-FRET species is ~39% and ~61%, which is almost equal to the population of conformational state 2 (~42%) and state 1 (~58%), revealed by the N103C-based smFRET characterization (Figure 3b and Figure S8b). Thus, for the D174C labeling site, the low- and high-FRET should account for state 2 and state 1, respectively. Indeed, titration experiments showed that state 1 is selectively enriched by the SAIRGA peptide (Figure S8b–e) and that state 2 is responsible for DNA recognition (Figure S8g–j). Similar to the smFRET results revealed by N103C dye-labeling, the SPbeta-derived GMPRGA peptide had no perturbation on the smFRET profile of phAimR (Figure S5b). The binding ability of phAimR to peptide and to DNA is 22.9 ± 0.5 nM and 1256.9 ± 217.6 nM in K_D_, respectively (Figure S8f,k). All of these smFRET results revealed by D174C dye-labeling are consistent with that of the N103C labeling site (Figure 3 and Figure S8).

## 4. Conclusions

**Figure 4:**
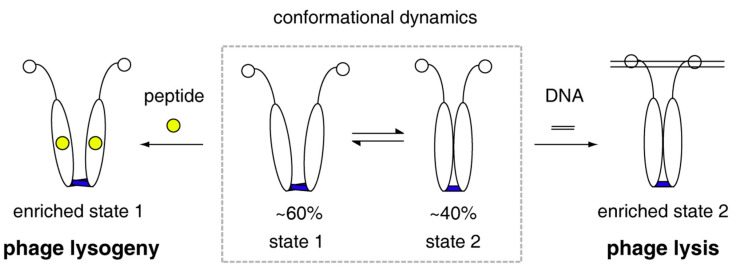
Model of phAimR dimer for target recognition in the lysis-lysogeny decisions of *Bacillus* phage phi3T. phAimR samples two interconverted conformational sates, and a specific preexisting state selectively recognizes the arbitrium peptide or the target DNA, respectively, for phage lysogeny or lysis.

Collectively, all the quantitatively biophysical characterizations of phAimR in solution and the revealed mechanism for specific target recognition allow us to propose a molecular model for the lysis-lysogeny decisions of *Bacillus* phage phi3T (Figure 4). The phAimR is a dynamic dimer and samples two distinct conformational states simultaneously, named state 1 and state 2. State 1 is responsible for arbitrium peptide recognition, leading to phage lysogeny, and state 2 selectively binds to target DNA promoting phage lysis. The intrinsic dynamics and inter-conversion of phAimR dimer between state 1 and state 2 enable phAimR to recognize a specific target via a conformational selection mechanism. This mechanism is different from the previously proposed model that the arbitrium peptide determines phage lysogeny by disassembling the phAimR dimer into a monomer [21]. The different conclusion may be due to the use of phAimR^Y341A/E371A^ for the reported crystallography studies [21]. Our previous studies on the target recognition of spAimR for the lysis-lysogeny decisions in *Bacillus* phage SPbeta have revealed that the spAimR also uses a conformational selection mechanism to recognize the arbitrium peptide and target DNA [24]. Thus, it seems that the strain-specific AimR proteins share a common mechanism for specific target recognition. The observation that different phages (phi3T and SPbeta) with different arbitrium peptides (SAIRGA and GMPRGA, respectively) influence lysis-lysogeny decisions to a different degree [10,21,25,26] could be due to a yet unknown strain-specific mechanism.

## Data Availability

Data is contained within the article, and are available on request from the corresponding author.

## Supplementary Materials

The following are available online at https://www.mdpi.com/article/10.3390/biom11091321/s1, Figure S1:Proposed strain-specific mechanism for the regulation of lysis-lysogeny decisions in SPbeta and phi3T phages, Figure S2: DNA oligonucleotides used for assessing smFRET parameters, Figure S3: Determination of the correction coefficient γ, Figure S4: AUC characterizations, Figure S5: The SPbeta-derived GMPRGA peptide has little perturbation on the phAimR smFRET profile, Figure S6: EMSA characterize the interaction between phAimR and target DNA, Figure S7: Comparison of cross-correlation function GAD(τ) between apo phAimR (black line), in complex with 500 nM SAIRGA peptide (blue line) and 8000 nM target DNA (red line), Figure S8: smFRET analysis of phAimR dimer for the recognition of SAIRGA peptide and target DNA.
