# Supplementary material for: AimR Adopts Preexisting Dimer Conformations for Specific Target Recognition in Lysis-Lysogeny Decisions of Bacillus Phage phi3T
Source: Biomolecules. 2021 Sep 7;11(9):1321. doi: 10.3390/biom11091321 (PMC8464984; doi:10.3390/biom11091321)
Supplement: Supplementary file 1 [file biomolecules-11-01321-s001.zip › biomolecules-1339680-supplementary.pdf]

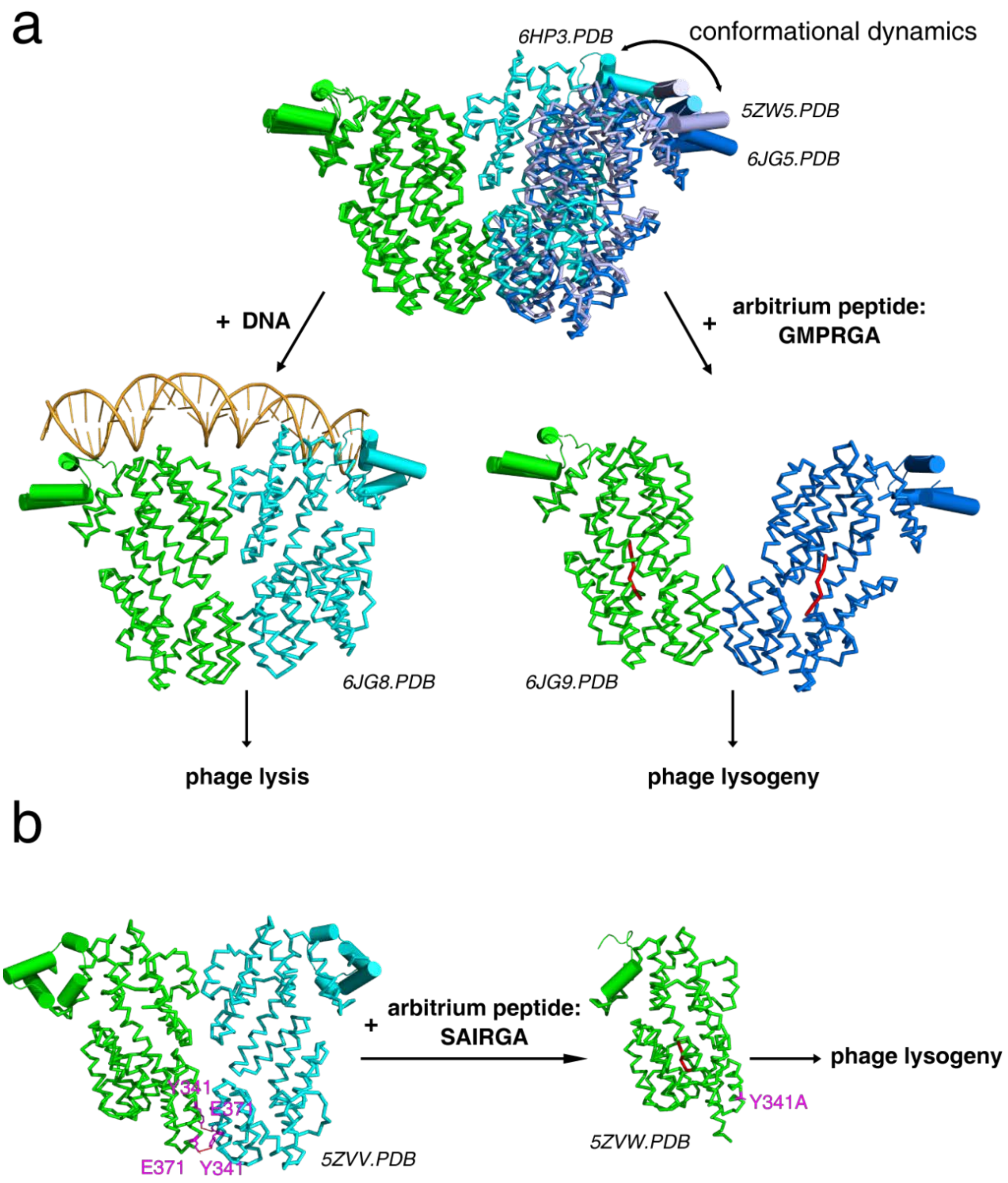

Figure S1. Proposed strain-specific mechanism for the regulation of lysis-lysogeny decisions in SPbeta and phi3T phages. (**a**) Crystallography studies for the regulation of lysis-lysogeny decisions in SPbeta phage. Multiple dimer conformations of spAimR were crystalized. The spAimR binds DNA in a closed dimer conformation and biases bacteriophages towards lysis, whereas it adopts an open dimer conformation to recognize the arbitrium peptide GMPRGA that causes phage lysogeny. (**b**) Crystallography studies for the regulation of lysis-lysogeny decision in phi3T phage. phAimR is dimeric and the phAimR$^{Y341A/E371A}$ mutant adopts a monomeric conformation to recognize the arbitrium peptide SAIRGA, leading to phage lysogeny. The complex structure of phAimR and DNA is still elusive. The PDB accession numbers of these structures are denoted. The arbitrium peptide and DNA in these structures are represented with red and yellow ribbon representation, respectively. The Y341, E371 and corresponding mutant residues of phAimR are represented in magenta sticks.

T: for Alexa488 labelling, T: for Cy5 labelling

T1B8:
5’-TAAATCTAAAGTAACATAAGGTAACATAACGGTAAGTCCA-3’
3’-ATTTAGATTTCATTGTATTCCATTGTATTGCCATTCAGGT-5’

T1B18:
5’-TAAATCTAAAGTAACATAAGGTAACATAACGGTAAGTCCA-3’
3’-ATTTAGATTTCATTGTATTCCATTGTATTGCCATTCAGGT-5’

D_only:
5’-TAAATCTAAAGTAACATAAGGTAACATAACGGTAAGTCCA-3’
3’-ATTTAGATTTCATTGTATTCCATTGTATTGCCATTCAGGT-5’

A_only:
5’-TAAATCTAAAGTAACATAAGGTAACATAACGGTAAGTCCA-3’
3’-ATTTAGATTTCATTGTATTCCATTGTATTGCCATTCAGGT-5’

Figure S2. DNA oligonucleotides used for assessing smFRET parameters. After annealing, double-stranded DNA oligonucleotides were prepared in a buffer of 20 mM Hepes (pH 7.0), 100 mM NaCl, 1 mM EDTA and 0.01% (vol/vol) Tween 20, with additional 1 mM L-ascorbic acid and 1 mM methylviologen[1]. The donor-only and acceptor-only DNA duplex samples were used to measure the cross-talk terms. The Lk term (donor emission leaked to the acceptor channel) was determined from the f_{DexAem}/f_{DexDem} ratio for the donor-only sample, and the Di term (direct excitation of the acceptor) was determined from the f_{DexAem}/f_{AexAem} ratio for the acceptor-only sample. The Lk term was determined at about 0.13, whereas the Di term was about 0.06 for the Alexa Fluor 488–Cy5 pair by our instrumentation. The doubly labeled duplex DNA sample with two donor–acceptor distances (separated by increasing the number of base pairs) was used to determine the correction coefficient γ (cf. Figure S3). The determination of Di and Lk values allows accurate determination of the FRET efficiency.

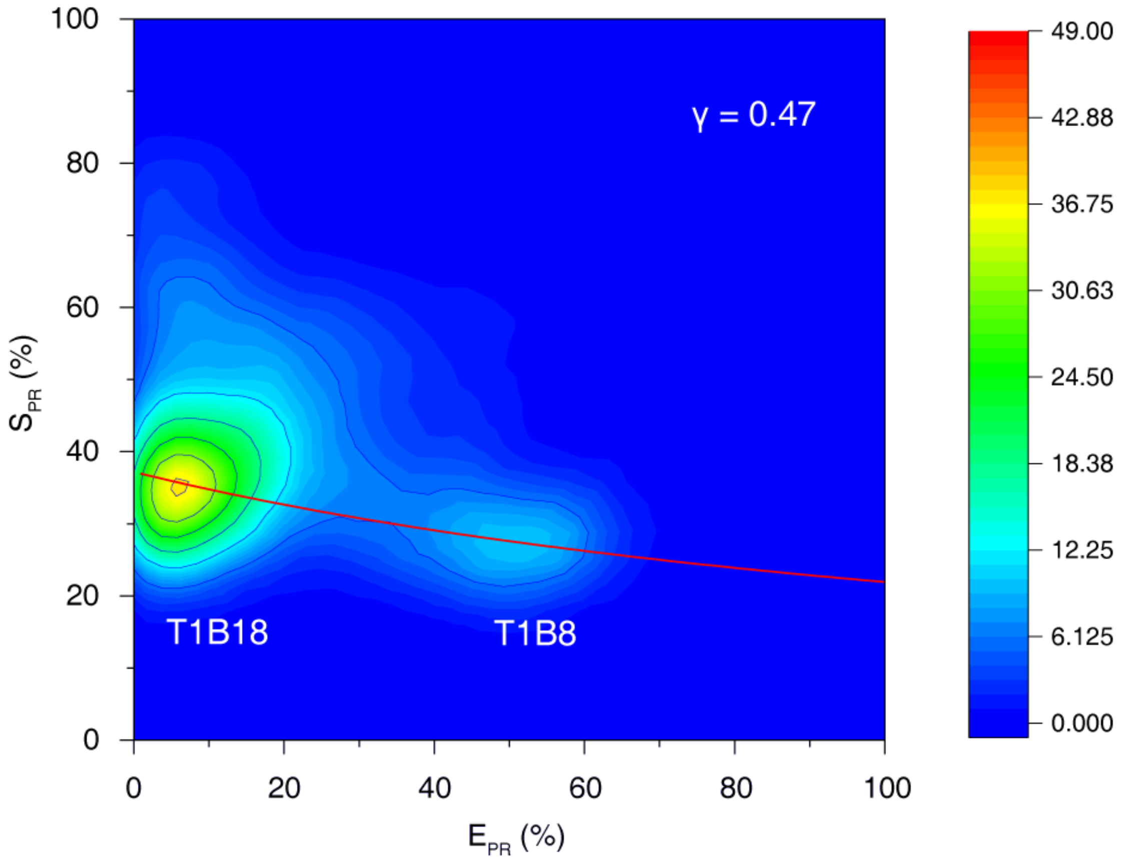

Figure S3. Determination of the correction coefficient γ. γ is defined as $\eta_{Aem}\varphi_A/\eta_{Dem}\varphi_D$, in which φ_A and φ_D are the quantum yields of the acceptor and donor fluorophores, respectively, and η_{Aem} and η_{Dem} are the detection efficiencies for the acceptor and donor channel detectors, respectively. Using the premixed T1B8 and T1B18 DNA samples shown in Figure 1S, we measured the FRET efficiencies and ensured > 3 counts per bin for photon time trace $f_{Aex/Aem}$ and > 4 counts per bin for photon time trace $f_{Dex/Dem}$, to remove donor-only and acceptor-only species. Two-dimensional heat maps were plotted for the cross-talk–corrected proximity FRET transfer efficiency ratio E_{PR} versus the cross-talk–corrected stoichiometry ratio S_{PR}. The S_{PR} is defined as $(f_{Dex/Dem} + f_{Dex/Aem} - Lk - Di)/(f_{Dex/Dem} + f_{Dex/Aem} + f_{Aex/Aem} - Lk - Di)$. Fitting the hottest bins of S_{PR} and E_{PR}, $1/S_{PR} = 1 + \gamma\beta + \beta(1-\gamma)E_{PR}$[2], we obtained the γ-value at 0.47 and the β-value at 3.56. Calibration of the γ allows accurate determination of the FRET efficiency.

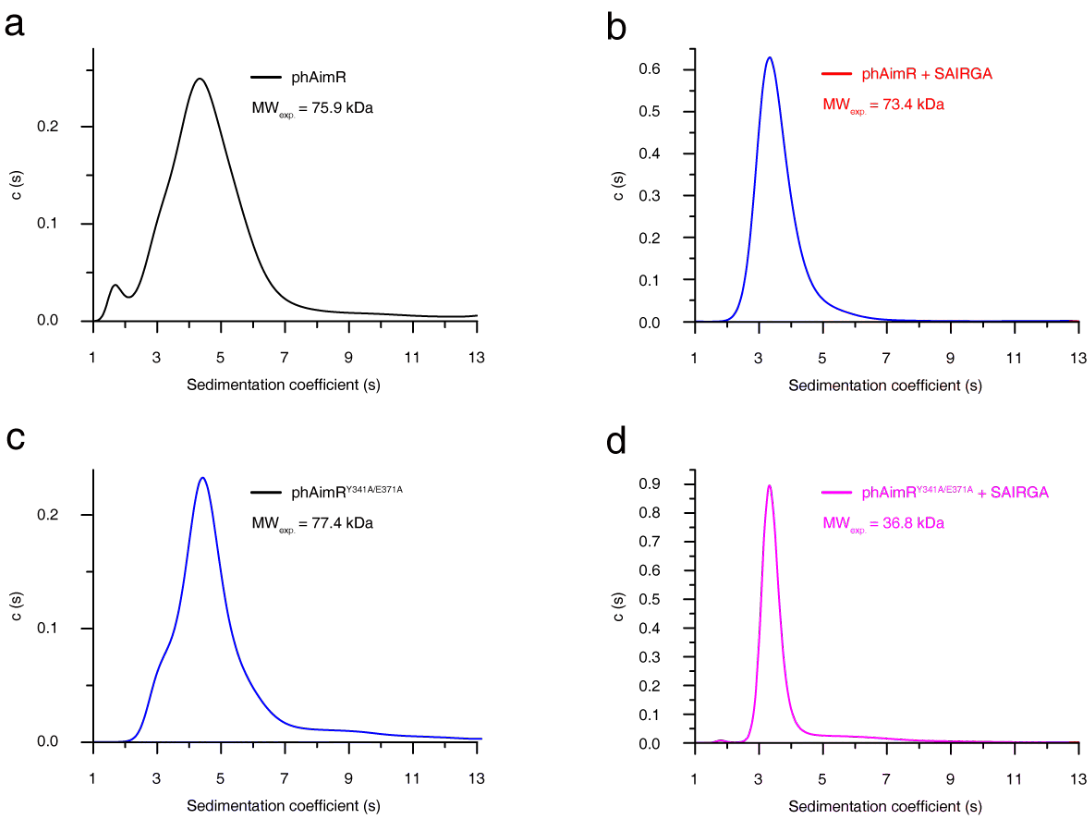

Figure S4. AUC characterizations. AUC experiments evaluate the experimental molecular weight of (**a**) phAimR, (**b**) phAimR in complex of SAIRGA peptide, (**c**) phAimR$^{Y341A/E371A}$ mutant, and (**d**) phAimR$^{Y341A/E371A}$ mutant in complex of SAIRGA peptide, respectively.

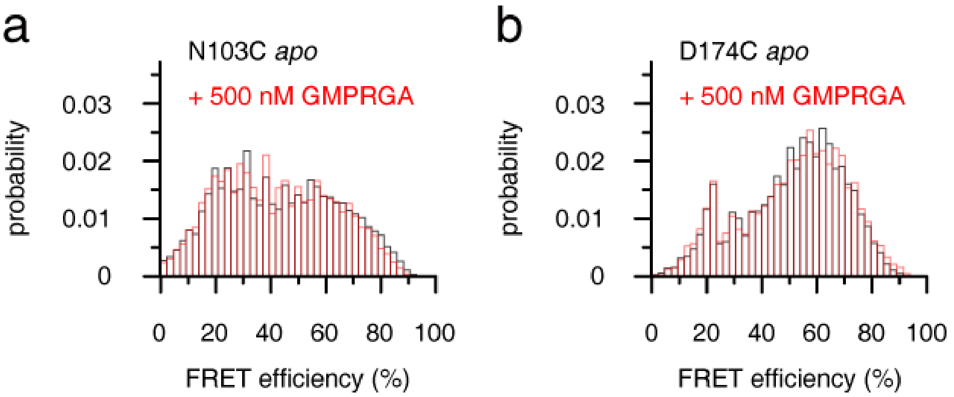

Figure S5. The SPbeta-derived GMPRGA peptide has little perturbation on the phAimR smFRET profile. (**a**) The N103C-observed smFRET profile of phAimR (black histogram) and in the presence of 500 nM GMPRGA peptide (red histogram). (**b**) The D174C-observed smFRET profile of phAimR (black histogram) and in the presence of 500 nM GMPRGA peptide (red histogram).

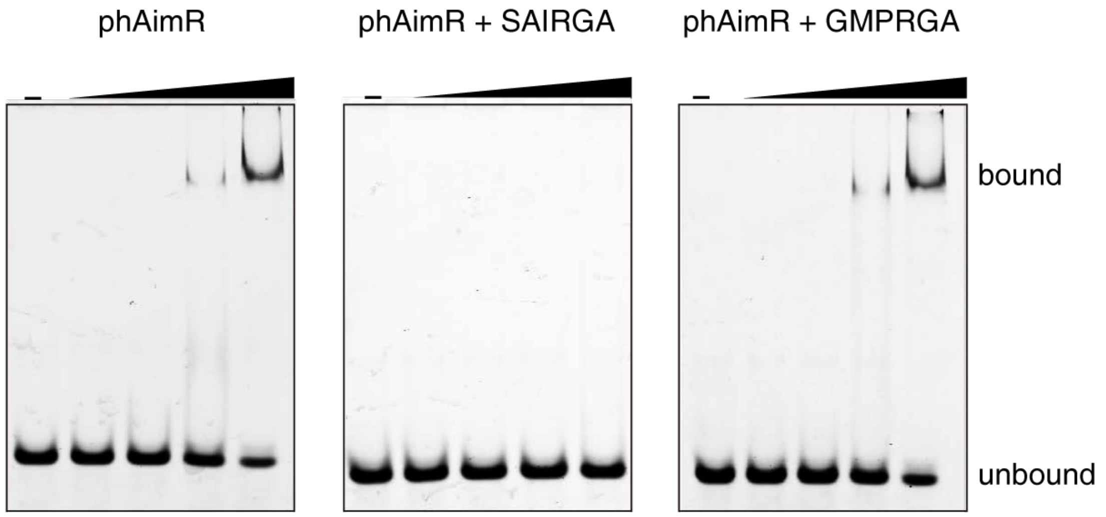

Figure S6. EMSA characterize the interaction between phAimR and target DNA. The phi3T-derived SAIRGA peptide abolishes the DNA binding ability of phAimR, whereas the SPbeta-derived GMPRGA peptide has no effects. Each EMSA experiments was conducted for three biological replicates.

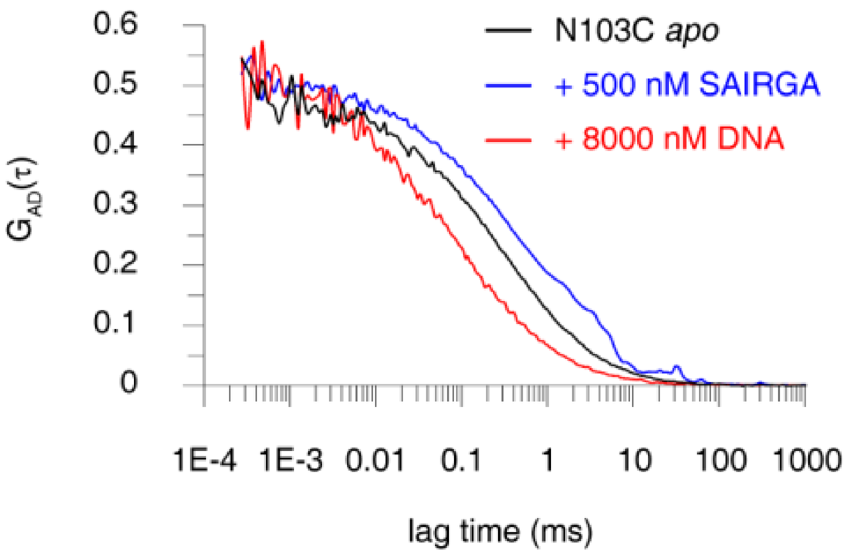

Figure S7. Comparison of cross-correlation function $G_{AD}(\tau)$ between *apo* phAimR (black line), in complex with 500 nM SAIRGA peptide (blue line) and 8000 nM target DNA (red line). FCS data was collected for the N103C dyes-labeled phAimR dimer.

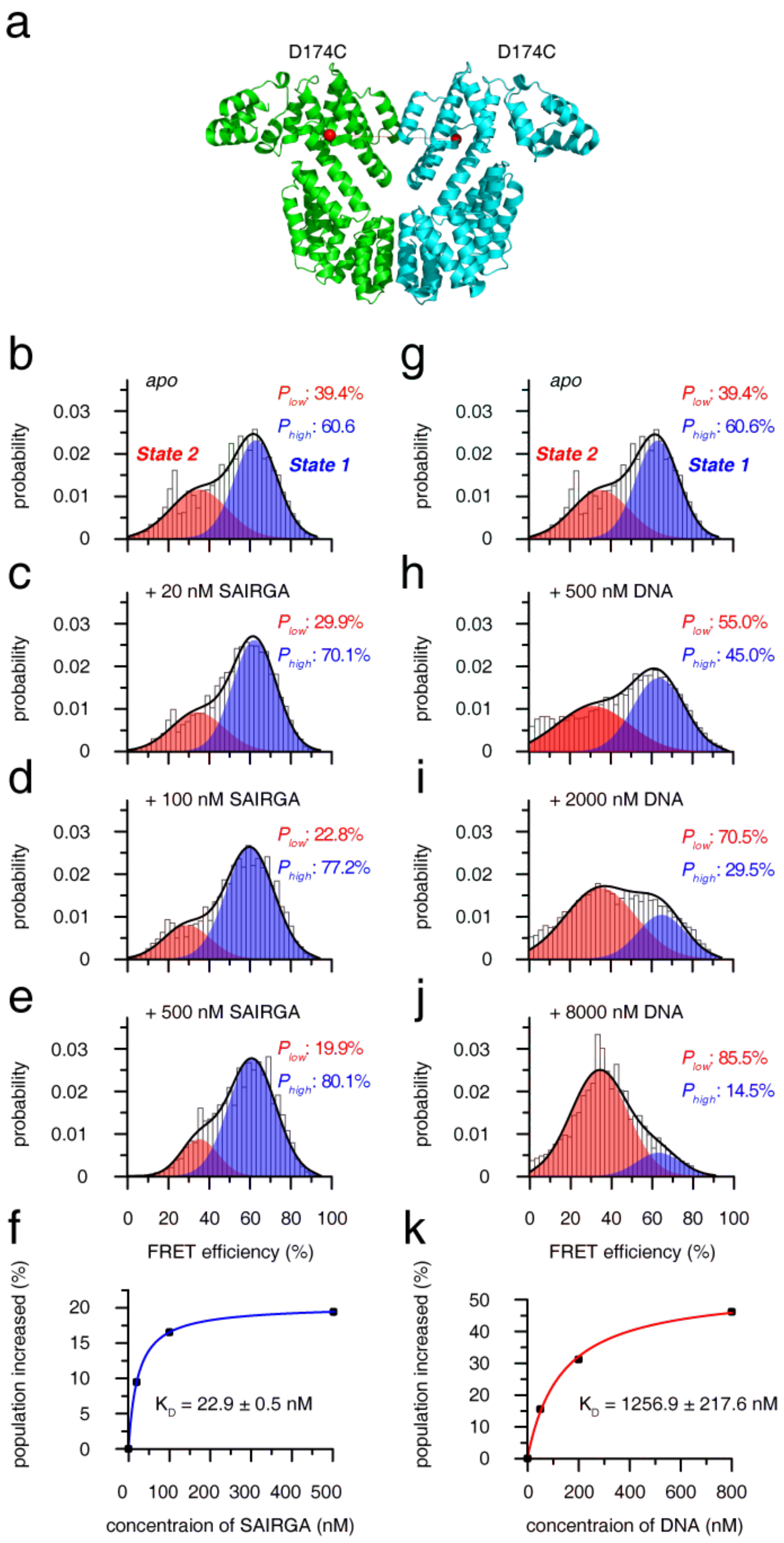

Figure S8. smFRET analysis of phAimR dimer for the recognition of SAIRGA peptide and target DNA. (**a**) Cartoon representation of phAimR dimer for the fluorophore conjugation site.

Structure with 5ZVV PDB code is used for the representation, and the Cα atoms of the D174 residues are shown as red spheres. (**b-e**) The Change of smFRET profile of phAimR dimer upon SAIRGA peptide titration. The low- and high-FRET species are colored in red and blue, respectively. The SAIRGA peptide selectively enriches the high-FRET species. (**f**) Binding affinity of phAimR towards SAIRGA peptide. (**g-j**) The Change of smFRET profile of phAimR dimer upon target DNA titration. The DNA selectively enriches the low-FRET species. (**k**) Binding affinity of phAimR towards DNA. Note the panels b and g are identical for easy comparison.

References

1. Vogelsang, J.; R. Kasper; C. Steinhauer; B. Person; M. Heilemann; M. Sauer; P. Tinnefeld; A reducing and oxidizing system minimizes photobleaching and blinking of fluorescent dyes, *Angew Chem Int Ed Engl*, 2008, 47(29):5465-5469.
2. Lee, N.K.; A.N. Kapanidis; Y. Wang; X. Michalet; J. Mukhopadhyay; R.H. Ebright; S. Weiss; Accurate FRET measurements within single diffusing biomolecules using alternating-laser excitation, *Biophys J*, 2005, 88(4):2939-2953.
